# Understanding the relationship between education-based hypergamy and women's social interaction in China: mediating roles of career aspirations and social distrust

**DOI:** 10.3389/fpubh.2025.1514794

**Published:** 2025-04-16

**Authors:** Penghui Wu, Ming Zhang, Senhao Xiong

**Affiliations:** ^1^Center for Studies of Education and Psychology of Ethnic Minorities in Southwest China, Southwest University, Chongqing, China; ^2^School of Education, Khon Kaen University, Khon Kaen, Thailand

**Keywords:** education-based hypergamy, social interaction, career aspirations, social distrust, CGSS 2021

## Abstract

**Background:**

Education-based hypergamy (EBH) is a key factor influencing the social interaction (SI) of women in China. Women in education-based hypergamous marriages, where they have lower education levels than their spouses, often face unique challenges that may impact their social lives. The dynamics of traditional gender norms and opportunity costs of balancing career and household duties present barriers to SI for these women. Thus, understanding the mechanisms that mediate and moderate this relationship is crucial for addressing social inequalities and improving public health outcomes.

**Methods:**

Data for this study were obtained from the 2021 Chinese General Social Survey (CGSS), which sampled 1,442 women. We employed a mediation effect testing model to examine the relationships among EBH, career aspirations (CA), social distrust (SD), and SI. The model also tested the moderating role of household income in mitigating the effects of EBH on SI.

**Results:**

This study first highlights that EBH is negatively associated with women's SI. Notably, household income moderated this relationship, with higher income levels alleviating the negative impact of EBH on SI. Additionally, CA and SD were found to mediate the relationship between EBH and SI, demonstrating that these factors contribute to the reduction in SI among women in education-based hypergamous marriages.

**Discussion/conclusion:**

These findings partially align with prior research on gender norms and social interaction, offering theoretical insights into the negative effects of EBH on women's SI. From a public health perspective, the results underscore the need for policies that enhance household income and provide flexible work arrangements. Such policies could reduce the constraints imposed by EBH, thereby promoting better social interaction, mental well-being, and overall health for women.

## 1 Introduction

Social interaction, defined as the process by which individuals act and react in relation to others, plays a pivotal role in fostering mental health, wellbeing, and overall life satisfaction. Social interactions are instrumental in building support networks, enhancing cognitive function, and providing emotional support, which collectively contribute to an individual's resilience and psychological health (1, 2). Given the positive effects of social interaction, numerous studies have reviewed the factors that can influence social interaction, including socioeconomic status (3), education (4), cultural norms (5), health (6), technology use (7), psychological factors (8), and residential environment (9).

Marriage significantly influences social interaction, often leading to improved financial stability and higher social status, which in turn enhances participation in social activities and community engagement (10, 11). Culturally, marriages may alter social identities and expectations, potentially leading to greater social mobility and changes in social circles (12).

However, the social dynamics within marriages characterized by educational disparities, such as education-based hypergamy, may lead to distinct outcomes for individual social interactions. Education-based hypergamy, where a woman has a lower level of educational attainment than her husband (13), presents unique constraints on women's social engagement. In such marriages, women may feel greater pressure to conform to traditional gender roles, limiting their participation in social activities and interactions. Unlike marriages where educational parity exists, education-based hypergamy may reinforce imbalances in social power, further restricting women's autonomy in social spaces.

Globally, education-based hypergamy is not unique to China; similar trends are observed in various societies, such as the United States, India, and south Korean (14–16). In these contexts, women in hypergamous marriages often face societal pressures and gendered expectations that restrict their social mobility and engagement, albeit shaped by different socio-economic structures and gender norms. In China, where rising educational attainment among women contrasts with deeply ingrained traditional gender roles, education-based hypergamy remains notably prevalent. Recent studies indicate that nearly 40% of Chinese women marry men with higher educational qualifications (17). This trend reflects broader structural factors, including persistent gender inequalities in education and traditional divisions of labor in households, where men are expected to manage external affairs, and women focus on domestic duties (18). These entrenched norms not only limit women's social autonomy but also perpetuate power imbalances within the household, restricting women's ability to cultivate active and diverse social networks.

This study aims to explore the impact of education-based hypergamy on women's social interaction in China, with a focus on the mediating roles of career aspirations and social distrust. These factors influence how women navigate the balance between domestic responsibilities and social engagement. Additionally, the study examines the moderating effect of household income, which may either amplify or mitigate the effects of education-based hypergamy on women's social lives.

Drawing on data from the 2021 Chinese General Social Survey (CGSS), this paper seeks to address the following research questions:

Does education-based hypergamy affect the social interaction of women in China?

How do social distrust and career aspirations mediate the relationship between education-based hypergamy and women's social interaction?

How does household income moderate the relationship between education-based hypergamy and women's social interaction?

The structure of the paper is as follows: the section titled “Theoretical Framework, and Research Hypotheses” reviews the existing literature on the relationship between education-based hypergamy and social interaction among women. The “Data and Methods” section explains the data and describes the empirical model used. In the “Results and Analysis” section, we present and interpret the main findings. Lastly, the “Discussion and Conclusion” section provides a discussion of the results and offers a summary.

## 2 Theoretical framework and research hypotheses

### 2.1 Education-based hypergamy

Education-based hypergamy refers to a marital arrangement where the woman has a lower level of educational attainment compared to her husband (13). This phenomenon is particularly relevant in societies where traditional gender roles and expectations persist, such as China (19). Some studies argue that these women in education-based hypergamy might face challenges in navigating new social circles, potentially feeling out of place or pressured to conform to higher social expectations, which can inhibit their social interactions (12, 20).

Power dependence theory and opportunity cost theory provide a comprehensive framework for understanding the relationship of education-based hypergamy and social interactions among women. According to power dependence theory, the educational imbalance in hypergamy often places women in a dependent position because their lower educational status typically translates to lesser bargaining power within the marriage, making them more reliant on their husbands for social and economic resources (21). Women may feel less entitled or capable of participating in social activities, particularly those involving higher-status social circles. The fear of not meeting the expectations of these social environments, coupled with a sense of inferiority, can lead to reduced social interaction (22). Furthermore, the opportunity cost theory posits that the time and energy women invest in domestic responsibilities, which are often exacerbated in Education-based hypergamy due to traditional role expectations, come at the expense of social interactions. The higher the educational and career aspirations of the husband, the greater the opportunity cost for the wife to engage in social activities, as she is expected to manage household duties more intensively (23). This trade-off significantly reduces the frequency and quality of social interactions for women in Education-based hypergamy, as they prioritize domestic and caregiving roles over social engagements.

Based these, we have hypothesis 1.

Hypothesis 1: Women in education-based hypergamy (women's education levels are lower than men's) have less social interaction frequency.

### 2.2 Social distrust

Societal expectations of individuals are shaped by their gender roles, social status, and other identity attributes (24). In the context of education-based hypergamy, women may develop social distrust due to perceived mismatches in status or cultural capital between themselves and others (25). Bourdieu (1986) emphasizes that education, as a form of cultural capital, plays a key role in shaping social status and access to networks, with those possessing higher levels of education typically occupying more privileged positions (26). In hypergamy, women with higher educational attainment than their husbands may perceive themselves as having lower social capital in comparison to their spouse or peers, leading to feelings of marginalization and undervaluation. This perceived discrepancy can trigger a higher level of social distrust, where women begin to question the motives and trustworthiness of others in social situations.

The Opportunity Cost Theory suggests that individuals often make decisions based on the trade-offs between the benefits and costs of available options (27). In the case of women in education-based hypergamous marriages, the “opportunity costs” of engaging in social interactions may appear too high if they feel socially devalued or inferior. These women may perceive social interactions as more costly than beneficial, given the potential emotional toll of feeling excluded or discriminated against due to their perceived mismatch in educational or social status. Thus, this theory helps explain why women in education-based hypergamous marriages may develop greater social distrust—because they see social interactions as potentially harmful, diminishing their overall social wellbeing.

Moreover, trust is a cornerstone of social engagement. When trust is lacking, individuals are less likely to engage in social interactions, as mutual trust and reciprocity are essential for meaningful social exchanges (28). Women experiencing social distrust, particularly as a result of their educational disparities, are more likely to withdraw from social activities to avoid potential rejection or discrimination. This withdrawal can reduce their social networks and hinder their participation in beneficial social support systems. Therefore, social distrust is hypothesized to serve as a mediating variable between education-based hypergamy and social interaction frequency.

Based on these insights, we propose the following hypotheses:

Hypothesis 2: Women in education-based hypergamy will exhibit greater social distrust toward others.
Hypothesis 3: Social distrust mediates the relationship between education-based hypergamy and the frequency of social interactions among women.

### 2.3 Career aspirations

Career aspirations refer to an individual's ambitions and goals regarding their professional development and advancement (29). In education-based hypergamy, women's career aspirations often face significant barriers. The husband's higher educational attainment typically brings greater financial stability, reducing the perceived necessity for the wife to seek or advance her career. This financial security, combined with traditional expectations that prioritize women's domestic roles, discourages women from pursuing professional ambitions (30, 31).

Power dependence theory suggests that women in education-based hypergamy may experience diminished bargaining power within the marriage due to their lower educational and economic status. This dependence often confines them primarily to domestic roles (32). Opportunity cost theory further explains that the economic returns from the wife's employment are often viewed as negligible compared to the perceived benefits of focusing on domestic responsibilities. This economic reasoning, reinforced by traditional gender norms, leads women to deprioritize their careers.

Additionally, the lack of career aspirations can mediate the relationship between education-based hypergamy and social interactions (33). Employment often provides women with social networks and opportunities outside the home (34, 35). Without a professional role, women have fewer social contacts and opportunities, leading to reduced social interaction (36, 37).

Based on these insights, we propose:

Hypothesis 4: Women in education-based hypergamy are less likely to pursue or advance their own careers.
Hypothesis 5: The lack of career aspirations mediates the relationship between education-based hypergamy and reduced social interaction.

### 2.4 Household income as a moderating role

Household income may play a critical moderating role in shaping women's social interaction patterns, particularly in the context of traditional domestic expectations reinforced by hypergamy (38). Women in higher-income households often have greater access to resources that enable them to maintain broader social networks, as financial stability can alleviate some of the pressures tied to household responsibilities, thus allowing for more social engagement (39). Higher household income can also provide women with opportunities to participate in social and professional activities that enhance their wellbeing, acting as a buffer against potential isolation in hypergamous marriages.

Research shows that higher household income is associated with increased social capital, which includes more diverse and extensive social networks (9, 40). This social capital can mitigate the negative effects of educational disparity in marriage by enabling women to maintain their frequency of social interactions. Furthermore, women in higher-income households may face less stigma and fewer constraints in participating in social activities, as financial security can lessen the pressure to conform strictly to traditional gender roles (41).

Given these perspectives, we propose:

Hypothesis 6: The negative impact of education-based hypergamy on social interaction is weaker for women in higher-income households.

**Figure 1:**
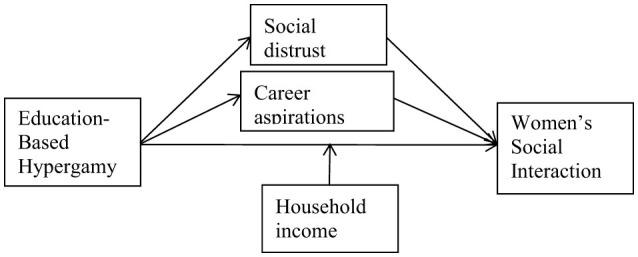
Conceptual model.

Here's a conceptual framework diagram illustrating the relationship between education-based hypergamy and the social interaction of women in China. The diagram highlights the moderating role of household income and the mediating mechanisms of social distrust and career aspirations. Our conceptual model is presented in Figure 1.

## 3 Data and methods

### 3.1 Data

The empirical analysis in this study is based on the representative dataset from the 2021 China General Social Survey (CGSS). Since its inception in 2003, the CGSS has been conducted by academic institutions and stands as the earliest national survey in mainland China to cover over 10,000 households. It includes comprehensive information on demographics, family structure, economic conditions, social attitudes, and statuses. Scholars have utilized CGSS as a crucial data resource for investigating social interactions. For instance, (42) some have employed it to analyze the impact of health on social interactions, while others have explored the relationship between social interaction and public pro-environmental behavior (43). The CGSS dataset contains extensive data on the social interactions of women, which is essential for replicability. It provides detailed insights into the social lives of women within the broader context of Chinese society.

The sample consists of women aged 30 to 50, a range chosen based on the centrality of marriage and career-related mediating variables. Women in this age group are more likely to have established careers and family roles, making them more relevant for analyzing social distrust and career aspirations as mediating variables. We specifically excluded men to ensure the study is aligned with the research focus on women. Furthermore, samples below 30 and above 50 were excluded, as they were deemed less likely to be directly involved in education-based hypergamy and the associated mediating factors. Lastly, any samples with missing or anomalous data were removed, leaving a final sample size of 1,442 women.

### 3.2 Variables and measurement

#### 3.2.1 Dependent variable: women's social interaction

The primary dependent variable in this study is social interaction. Social interaction has been measured in previous research using various metrics, such as network size, density, diversity, and quality, as well as the proportion of expenses on personal relationships. However, consistent with the assumptions of our theoretical model, this study focuses on the frequency of social interactions as the key indicator.

To operationalize social interaction, we selected five questions from the CGSS 2017 questionnaire, which specifically assess the frequency of social interactions in the past year. These questions cover both formal and informal social activities that capture a broad range of interactions, including meetings with relatives, friends, neighbors, and engaging in social entertainment. The selected questions are as follows:

“In the past year, have you often engaged in the following activities during your free time: meeting with relatives who do not live with you?”

“In the past year, have you often engaged in the following activities during your free time: meeting with friends?”

“In the past year, have you often engaged in the following activities during your free time: social visiting?”

“How often do you engage in social entertainment activities (e.g., visiting each other, watching TV, eating, playing cards, etc.) with your neighbors?”

“How often do you engage in social entertainment activities (e.g., visiting each other, watching TV, eating, playing cards, etc.) with other friends?”

Each of these questions is answered on a 5-point or 7-point Likert scale, ranging from low to high frequency. The average score across these five items is then used to determine an individual's overall social interaction frequency.

The choice of these specific questions was motivated by their ability to capture a wide range of social interactions across various settings—both private (family and friends) and public (neighbors, social entertainment). These activities reflect different aspects of social engagement, ranging from close relationships to more extended social networks. This comprehensive approach ensures a nuanced measure of social interaction frequency, which aligns with the conceptualization of social engagement in the theoretical framework of the study. Additionally, this measurement method has been used in previous studies, supporting its reliability and validity (44).

By focusing on frequency, this study captures how often individuals engage in social interactions, which is central to understanding the dynamics of social engagement in the context of education-based hypergamy.

#### 3.2.2 Independent variable: education-based hypergamy

The primary independent variable is education-based hypergamy. We first created one variable to measure educational attainment, divided into three levels: elementary education (1 = less than 6 years), secondary education (2 = 6–12 years), and higher education (3 = more than 12 years). We then defined hypergamy of education respectively. Education-based hypergamy is then measured by comparing the education levels of the husband and wife. If the wife's education level is lower than the husband's, the variable is coded as 1 (indicating hypergamy). If the wife's education level is equal to or higher than the husband's, the variable is coded as 0. For example, in a family where the wife has primary education and the husband has secondary education, the education-based hypergamy value is 1. This measurement method has also been used in other studies (45).

To ensure the robustness of the research findings, this study introduces a second indicator to measure the dependent variable, education-based hypergamy. Using the number of years of education as the basic unit, the marriage is classified as education-based hypergamy when the husband has more years of education than the wife, assigning it a value of 1. Conversely, when the husband's years of education are equal to or lower than the wife's, the value is set to 0. This alternative measurement provides a more nuanced understanding of educational disparities within marriage, ensuring the findings are not dependent on a single approach and offering deeper insights into the impact of these disparities on social interactions and subjective class identification.

#### 3.2.3 Mediating variables: social distrust and career aspirations

Career aspirations is assessed through responses to the question, “Which of the following best describes your current employment situation?” Responses are coded as follows: Value 0: “Business owner”, “Self-employed”, “Employed by others”, and “Temporary worker/Contract labor”. Value 1: “Casual worker,” “Working/helping in family business (paid),” “Working/helping in family business (unpaid),” and “Freelancer.” Social distrust is assessed through responses to the question, “Generally speaking, do you agree that most people in this society can be trusted?” Responses are given on a 5-point Likert scale, and the scores are reverse-coded by us, with higher scores indicating greater social distrust.

#### 3.2.4 Moderator variable: household income

Household income serves as the moderator variable in this study, operationalized through the survey question A62: “What was your household's total income for the entire year of 2020?” To assess the income level of the survey sample, this study utilizes the logarithmic value of last year's income. Although using this year's household income data would provide a more accurate reflection of current financial status, data limitations restrict access to such information. Given that personal annual income is a continuous variable, we believe that last year's income level can effectively serve as a proxy for the current income level.

#### 3.2.5 Control variables

The analysis controls for several demographic and socio-economic variables to ensure that the relationship between education-based hypergamy and social interaction frequency is not confounded by other factors. These control variables include:

Age: As a key factor influencing social engagement, age is likely to affect both social interaction patterns and the experience of education-based hypergamy. Older individuals may have different social interaction behaviors due to life stage differences (46).

Ethnicity: Ethnicity can play a significant role in shaping social networks and experiences. Different ethnic groups may have distinct cultural practices, expectations, and social engagement patterns, which could impact how education-based hypergamy affects social interactions. Including ethnicity as a control variable helps to account for these variations (47).

Health Condition: Health is a well-established determinant of social participation. Women with poor health may be less likely to engage in social activities, regardless of their marital or educational status. Therefore, controlling for health ensures that any observed relationship between education-based hypergamy and social interaction is not influenced by health-related limitations (48).

Political Identity: Political affiliation is included because it may correlate with broader social engagement and participation. For instance, party members may have different social expectations and engagement patterns compared to non-party members, potentially influencing their social interaction behavior. Controlling for political identity helps isolate the effects of education-based hypergamy from political differences (49).

Marriage Status: Given that marriage status directly affects social roles, including whether the individual is married or not, it is essential to control for this factor to ensure that the observed effects are not confounded by variations in marital status (50). The descriptive statistics for each of these variables are provided in Table 1.

**Table 1:** The descriptive statistics and definition of variables.

**Variable**	**Description**	**Obs**	**Mean**	**Std. dev**.	**Min**	**Max**
Social interaction	Average score of likert-scale responses on social interaction activities	1,442	2.99348	0.9639	1	5
Education based hypergamy	Index two	1,442	0.29404	0.4557	0	1
	Index one	1,442	0.32316	0.4678	0	1
Age	Years	1,442	38.7309	7.3641	20	50
Ethnicity	“Han ethnicity is coded as 1; other ethnic minorities are coded as 0.”	1,442	0.91193	0.2835	0	1
Politic	“Communist party members are coded as 1; others are coded as 0.”	1,442	0.07074	0.2564	0	1
Health	(Scale from 1 to 5)	1,442	3.85992	2.6497	1	5
Marriage	“Married is coded as 1; unmarried is coded as 0.”	1,442	0.9590	0.1983	0	1
Income	Household income (thousand yuan)	1,442	12.0962	2.0850	6.68461	16.1181
Social distrust	(Scale from 1 to 5)	1,442	2.5482	1.0102	1	5
Career aspirations	0 if employed (business owner, self-employed, employed by others, temporary worker/contract labor), 1 otherwise	1,442	0.68	0.4723	0	1

### 3.3 Model selection

We use a multiple linear regression model to conduct the empirical analysis. The model is set as follows:


(1) $$\begin{array}{l} Interaction_{i}=\beta_{0}+\beta_{1}Hypergamy_{i}+\beta_{3}Income_{i}\\ \;*Hypergamy_{i}+Control_{1}+\varepsilon_{1} \end{array}$$

Where Interaction_*i*_ denotes the social interaction of women *i*, it is a continuous numerical variable, Hypergamy_*i*_ is the independent variable, Income_*i*_ is the household income of women *i*, The interaction term Income_*i*_ * Hypergamy_*i*_ is used to test the moderating effect of household income and Control_*i*_ represents a series of control variables, including age, ethnicity, politic, health and marriage status. β_0_ is a constant term, β_1_ is the slope of Hypergamy_*i*_, β_2_ is the slope of Income_*i*_, β_3_ is the slope of Education_*i*_ * Hypergamy_*i*_ and ε_*i*_ is the possible error term.

## 4 Results and analysis

### 4.1 Results of the multiple regression analysis

In this paper, multiple regression analysis is employed to construct the model, as the dependent variable, social interaction, is a continuous variable. Prior to conducting the regression analysis, it is necessary to perform a multicollinearity test on the selected variables to ensure that the results are not adversely affected by multicollinearity. The results of the multicollinearity test indicate that the VIF values are all below 5, which is significantly lower than the threshold of 10, suggesting that there is no apparent collinearity in any of the models. Additionally, due to the varying scales of the independent variables, standardization is required to ensure the stability of the results.

The results presented in Table 2 show the impact of education-based hypergamy on the frequency of social interactions among women. The regression coefficients for education-based hypergamy consistently show a negative relationship with social interaction across all models. Specifically, in Column 1, the coefficient for education-based hypergamy is −0.1468 (*p* < 0.05), suggesting that for each unit increase in education-based hypergamy, social interaction decreases by 0.1468 points on a 5-point scale. In Column 2, this effect remains negative at −0.1386 (*p* < 0.05), and in Column 3, the coefficient is −0.1305 (*p* < 0.05), indicating a small but consistent negative impact on social interaction, even after controlling for sociodemographic variables like age, ethnicity, and political affiliation.

**Table 2:** Impacts of education-based hypergamy on social interactions.

**Variable**	**(1)**	**(2)**	**(3)**	**(4)**	**(5)**	**(6)**

	**Social interaction**	**Social interaction**	**Social interaction**	**Social interaction**	**Social interaction**	**Social interaction**
Hypergamy	−0.1468^**^	−0.1386^**^	−0.1305^**^	−0.1387^**^	−0.1356^**^	−0.1290^**^
	(0.0597)	(0.0598)	(0.0596)	(0.0582)	(0.0580)	(0.0577)
Age		−0.0147^***^	−0.0121^***^		−0.0150^***^	−0.0124^***^
		(0.0038)	(0.0038)		(0.0038)	(0.0038)
Ethnicity		0.3071^***^	0.2466^***^		0.3065^***^	0.2461^***^
		(0.0941)	(0.0951)		(0.0941)	(0.0951)
Politic		−0.1507	−0.2241^**^		−0.1455	−0.2196^**^
		(0.1012)	(0.1015)		(0.1009)	(0.1013)
Health		0.0182^*^	0.0157^*^		0.0182^*^	0.0158^*^
		(0.0094)	(0.0093)		(0.0094)	(0.0093)
Marriage			−0.1752^*^			−0.1766^*^
			(0.0936)			(0.0936)
Income			0.1217^***^			0.1217^***^
			(0.0290)			(0.0290)
Cons	3.0348^***^	3.2674^***^	2.3969^***^	3.0367^***^	3.2821^***^	2.4155^***^
	(0.0329)	(0.1737)	(0.4806)	(0.0336)	(0.1738)	(0.4809)
*N*	1442	1442	1442	1442	1442	1442
*R* ^2^	0.0051	0.0312	0.0493	0.0048	0.0313	0.0495

^***^, ^**^ and ^*^ denote significance at 1%, 5%, and 10% levels, and the number in the lower bracket is standard error.

Although the effects are statistically significant (*p* < 0.05), it is important to consider the practical implications of these findings. The magnitudes of the coefficients, while statistically significant, suggest that the negative relationship between education-based hypergamy and social interaction, while real, is relatively modest. For example, the impact of education-based hypergamy (−0.1305) implies that the difference in social interaction frequency between women with higher education-based hypergamy and those without is small, though it still suggests a meaningful pattern in terms of social behavior.

Furthermore, in the robustness tests using a different proxy for education-based hypergamy (Columns 4–6), we observe consistent negative effects, though the magnitude of the coefficients slightly decreases. This consistency strengthens the reliability of the findings, confirming that education-based hypergamy is associated with a reduced frequency of social interactions across different measures of the variable.

In practical terms, while the effect sizes are not large, the consistent negative association suggests that women with higher levels of education-based hypergamy may have slightly fewer social interactions than those without it, a factor that could be influenced by increased career focus or social positioning.

### 4.2 Testing for mediation effects of career aspirations and social distrust

We used the bootstrap method to verify the total, direct, and indirect effects. As shown in Table 3. The results from the bootstrap analysis with 1,000 iterations reveal the significant mediating roles of career aspirations and social distrust in the relationship between education-based hypergamy and social interaction. For social distrust, the direct effect of hypergamy on social interaction is estimated at −0.0165 [95% CI: (−0.03096, −0.00516)], accounting for 11.81% of the total effect, while the indirect effect through social distrust is −0.1232 [95% CI: (−0.22360, −0.01648)], representing 88.19% of the total effect. Similarly, for career aspirations, the direct effect is −0.0126 [95% CI: (−0.02623, −0.00214)], contributing 9.02% of the total effect, and the indirect effect through career aspirations is −0.1271 [95% CI: (−0.24518, −0.02639)], accounting for 90.98% of the total effect. These findings underscore the dominant role of both mediators in the negative impact of education-based hypergamy on social interaction. Similar to the baseline regression, to ensure the robustness of the research results, the study also used index two to measure education-based hypergamy. The results obtained are similar to those when using index two to measure education-based hypergamy.

**Table 3:** Bootstrap test results for multiple intermediary models.

**Variable**	**Measurement of independent variables**	**Effect**	**Effect value**	**95% Confidence interval**	**Proportion**
Distrust	Index one	Direct effect	−0.01650501	[−0.0309606, −0.0051609]	11.81%
		Indirect effect	−0.12321048	[−0.2235987, −0.0164812]	88.19%
	Index two	Direct effect	−0.01228254	[−0.0294787, −0.0023834]	9.56%
		Indirect effect	−0.11620579	[−0.2097813, −0.0058389]	90.44%
Work	Index one	Direct effect	−0.01260336	[−0.0262288, −0.0021419]	9.02%
		Indirect effect	−0.12711213	[−0.2451828, −0.0263866]	90.98%
	Index two	Direct effect	−0.00879663	[−0.0222466, −0.0012577]	6.85%
		Indirect effect	−0.11969171	[−0.2142328, −0.0069708]	93.15%

These results underline the practical significance of the mediating variables. While the direct effects of education-based hypergamy on social interaction are relatively small, the indirect effects mediated by career aspirations and social distrust are substantial. The dominance of these mediating effects suggests that the reduction in social interaction due to education-based hypergamy is largely driven by changes in personal attitudes toward work and trust in social networks, rather than by direct educational differences alone.

The findings also suggest that interventions targeting career aspirations and social trust could potentially mitigate the negative impact of education-based hypergamy on social interaction. For instance, fostering stronger social networks or encouraging work-life balance may help individuals with higher education-based hypergamy maintain social ties despite their career-focused orientation.

### 4.3 Testing the moderating effect model of household income

Table 4 presents the results of testing the moderating effect of household income on the relationship between education-based hypergamy and social interaction. In Column 2, the interaction term between income and hypergamy (income#hypergamy) is statistically significant (β = 0.1165, *p* < 0.10), suggesting that household income plays a significant moderating role in the relationship between education-based hypergamy and social interaction. Specifically, this interaction term indicates that higher household income weakens the negative association between education-based hypergamy and social interaction. In other words, individuals with higher household income experience less of a decline in social interaction due to higher education-based hypergamy, compared to those with lower household income.

**Table 4:** Testing the moderating effect of household income.

**Variable**	**(1)**	**(2)**	**(3)**	**(4)**	**(5)**	**(6)**
	**Social interaction**	**Social interaction**	**Social interaction**	**Social interaction**	**Social interaction**	**Social interaction**
Hypergamy	−0.1249^**^	−1.4257^**^	−1.4306^**^	−0.1202^**^	−1.2984^*^	−1.3016^*^
	(0.0594)	(0.6899)	(0.6849)	(0.0579)	(0.6779)	(0.6724)
Income	0.1409^***^	0.1062^***^	0.0873^**^	0.1415^***^	0.1080^***^	0.0880^**^
	(0.0282)	(0.0336)	(0.0344)	(0.0282)	(0.0341)	(0.0348)
Income#hypergamy		0.1165^*^	0.1138^*^		0.1054^*^	0.1049^*^
		(0.0616)	(0.0611)		(0.0604)	(0.0599)
Age			−0.0120^***^			−0.0123^***^
			(0.0038)			(0.0038)
Ethnicity			0.2476^***^			0.2462^***^
			(0.0950)			(0.0950)
Politic			−0.2134^**^			−0.2131^**^
			(0.1016)			(0.1012)
Health			0.0157^*^			0.0158^*^
			(0.0093)			(0.0093)
Marriage			−0.1841^**^			−0.1852^**^
			(0.0936)			(0.0936)
Cons	1.4474^***^	1.8369^***^	2.8059^***^	1.4426^***^	1.8190^***^	2.8160^***^
	(0.3184)	(0.3789)	(0.5280)	(0.3182)	(0.3843)	(0.5321)
*N*	1442	1442	1442	1442	1442	1442
*R* ^2^	0.0258	0.0288	0.0522	0.0257	0.0283	0.0520

^***^, ^**^ and ^*^ denote significance at 1%, 5%, and 10% levels, and the number in the lower bracket is standard error.

Additionally, the coefficients for education-based hypergamy (β = −1.4257, p <0.05) and household income (β = 0.1062, *p* < 0.01) remain significant, indicating that both variables independently influence social interaction. Education-based hypergamy continues to exert a significant negative effect on social interaction, while household income has a positive and significant impact, suggesting that individuals with higher income are more likely to maintain or enhance their social interaction, even in the presence of higher education-based hypergamy.

Column 3 includes additional control variables, such as age, ethnicity, political affiliation, health, and marital status, to ensure the robustness of the findings. The results are consistent with those in Column 2, confirming the stability of the moderating effect of household income. The inclusion of control variables strengthens our confidence in the validity of the findings. Columns 4–6 present the results when using index two to measure education-based hypergamy. The results remain largely similar to those obtained when using index one, further ensuring the robustness of our research findings. Specifically, the moderating effect of household income is consistent across both indices, reinforcing the argument that income plays a key role in moderating the negative impact of education-based hypergamy on social interaction.

The findings have important implications for understanding how socioeconomic factors influence social behaviors. While education-based hypergamy generally leads to decreased social interaction, particularly due to greater social stratification, individuals with higher household incomes seem to be somewhat protected from this negative effect. This suggests that household income may act as a buffer, allowing individuals with higher education-based hypergamy to maintain social networks despite the challenges posed by educational differences.

Interventions aimed at enhancing income equality or supporting individuals from lower-income households may help mitigate the negative effects of education-based hypergamy on social interaction. For example, policies promoting social mobility or improving access to resources could alleviate some of the social isolation associated with educational disparities.

### 4.4 Endogenous discussion and robustness test

We recognize that the results of the baseline analysis need to consider the potential issue of endogenous selection bias. The independent variable education-based hypergamy may be endogenous because the probability of women choosing this type of marriage may not be random and could be influenced by various factors. Therefore, we employed the Propensity Score Matching (PSM) method to address potential endogenous selection bias and obtain more reliable empirical results.

PSM works by matching individuals who have received the treatment (in this case, women who have chosen education-based hypergamy) with those who have not, based on a propensity score. The propensity score is the probability of receiving the treatment given a set of observed covariates. This score is typically estimated using a logistic regression model.

The primary goal of PSM is to create a sample of treated and untreated individuals that are comparable on the covariates, thus mimicking a randomized controlled trial. This allows for a more accurate estimation of the Average Treatment Effect (ATE).

Estimate Propensity Scores: The first step is to estimate the propensity score, *P(X)*, which is the probability of a woman choosing education-based hypergamy given her characteristics *X*. This is done using a logistic regression model:


$$\begin{array}{l} P\left(X\right)=P\left(W=1/X\right)=E\left[E|X\right] \end{array}$$

Here, is a binary variable where if the woman chooses education-based hypergamy, and otherwise.

Match Individuals: Once the propensity scores are estimated, individuals who chose education-based hypergamy (treated group) are matched with individuals who did not (control group) based on their propensity scores. This can be done using various matching algorithms such as nearest neighbor matching, caliper matching, or kernel matching.

Estimate the Treatment Effect: After matching, the average treatment effect (ATE) is estimated by comparing the outcomes of the treated and control groups. The ATE is given by:


$$\begin{array}{l} ATE=E\left\{\left[\left(Y_{1i}|W_{i}=1\right)-\left(Y_{0i}|W_{i}=0\right)\right]|X_{i}\right\} \end{array}$$

Here, *Y*_1*i*_ and *Y*_0*i*_ represent the social interaction for the same individual *i* under the conditions of choosing and not choosing education-based hypergamy, respectively. Since *Y*_1*i*_ and *Y*_0*i*_ cannot be observed simultaneously, matching helps in approximating these values.

First, we estimate the propensity score by a logistic model based on covariates including Age, education, ethnicity, political affiliation, personal gender cultural norms, family economic status at age 14, and household registration status at age 14. Then, we calculate the average treatment effects on the treated (ATT) of hypergamy_dummy based on the treatment (1) and control groups (0). Table 5 presents the empirical results of PSM using three matching methods, showcasing that the results of ATE remain significant and the signs of the results are negative.

**Table 5:** The results of propensity score matching (PSM) analysis.

**Matching method**	**Treatment group**	**Control group**	**Average treatment effect (ATT)**	**Standard error (SE)**	***T*-value**
Nearest Neighbor matching (*k* = 4)	4.47761	4.66273	−0.18512^**^	0.075	2.47
Radius matching	4.48922	4.66256	−0.17334^**^	0.069	2.51
Kernel matching	4.47761	4.66917	−0.19156^**^	0.072	2.66

^***^, ^**^ and ^*^ denote significance at 1%, 5%, and 10% levels.

In Table 5, we applied three different matching methods (nearest neighbor matching, radius matching, and kernel matching) for the propensity score matching analysis. The results indicate that regardless of the matching method used, education-based hypergamy has a negative and statistically significant impact on social interaction frequency. This suggests that even when considering potential endogenous selection bias, education-based hypergamy significantly reduces the social interaction frequency of women.

In the previous section, we addressed the self-selection issue of educational hypergamy using propensity score matching (PSM). Although we selected numerous variables to mitigate bias as much as possible, there may still be unobserved factors influencing women's educational hypergamy. This limits the validity of the results. To address this, we plan to use instrumental variables to strengthen causal inference. Table 6 is the results of instrumental variable regressions.

**Table 6:** The results of instrumental variable regressions.

**Variable**	**(1) First stage**	**(2) Second stage**
Educational		−0.088^***^
(*t*-value)		−2.24
Instrumental variable	−1.423^***^	
(*z*-value)	(−2.41)	
Individual control variables	Controlled	Controlled
Village control variables	Controlled	Controlled
Constant	3.417^***^	2.729^***^
(*t*-value)	−19.18	−22.54
Sample Size	1,442	1,442
Adj. *R*^2^	0.0425	0.0436
*F*-statistics	17.5956	

^***^, ^**^ and ^*^ denote significance at 1%, 5%, and 10% levels.

The educational background of parents can affect their children's marriage choices. Parental educational background can influence children's perceptions and preferences regarding marriage. Well-educated parents tend to have more open-minded views on marriage and may encourage their children to choose partners who are more “matched” in terms of career, economic status, and education levels. Conversely, less-educated parents may lean toward traditional views on marriage, placing greater emphasis on practical factors like economic stability (51).Furthermore, studies indicate that a mother's educational attainment is more likely to affect offspring compared to a father's education (52). Based on this, we will use the mother's educational level as an instrumental variable to examine the impact of educational hypergamy on women's social interactions.

The mother's educational attainment is strongly correlated with whether the offspring choose educational hypergamy, but it does not have a direct impact on social interactions. Therefore, the selection of the instrumental variable satisfies the conditions of relevance and exogeneity. The first-stage regression results show that the mother's educational level has a significant negative impact on educational hypergamy. The second-stage instrumental variable estimation results indicate that after addressing the endogeneity issue, educational hypergamy remains negatively correlated with social interactions. Additionally, the *F*-test value is 17.5956, >10, which demonstrates the validity of the instrumental variable selection. This also verifies the robustness of the baseline results.

## 5 Discussion and conclusion

Drawing from Power Dependence Theory and Opportunity Cost Theory, the current study examined the relationships between education-based hypergamy, career aspirations, social distrust, and social interactions. A research-based conceptual model was developed and tested using multiple linear regression and mediation analysis, with results largely supporting the proposed hypotheses.

Firstly, As hypothesized, education-based hypergamy negatively influenced women's social interactions, consistent with existing literature (53). China, as part of the Confucian cultural sphere, is deeply influenced by traditional gender norms (54). Women who are at an educational disadvantage tend to return to traditional household roles. However, modernization has prompted many women to seek social engagement outside the home (55). Although the rise in higher education among women in China (56) is decreasing the prevalence of education-based hypergamy, those in such marriages still experience reduced social interaction. This finding resonates with global discussions on how gender roles, shaped by educational disparities, impact women's social engagement, and highlights the broader relevance of this issue in the public health literature, especially in societies experiencing rapid modernization.

Secondly, from the perspective of the mediation mechanism, both career aspirations and social distrust significantly influence the social interactions of married women. Career aspirations, as a mediating factor, reflect how educational disparities in marriage affect women's professional ambitions. In hypergamy, where the woman's educational level is lower than her husband's, the woman's career aspirations may be suppressed due to traditional gender roles, family responsibilities, and societal expectations, which are emphasized by Confucian values in China. Deep-rooted cultural norms often prioritize women's domestic roles over their professional growth (57). Additionally, social distrust plays a crucial mediating role by illustrating how women in education-based hypergamy may develop feelings of distrust toward others. In China's hierarchical society, disparities in educational attainment can influence social interactions, with women in hypergamy potentially feeling marginalized or undervalued in social settings (58). This is particularly true when their spouse holds greater cultural capital, creating a sense of inferiority that fuels distrust toward others. Given that trust is foundational for social relationships, an increase in social distrust can lead to a reduction in social engagement. These insights align with global public health research that emphasizes the detrimental effects of social distrust on mental health and social wellbeing.

Thirdly, the results indicate that household income moderates the negative impact of education-based hypergamy on social interactions. Women with higher household income tend to have more extensive and diverse social networks (59). This can be explained by the relationship between economic resources and social capital, as (51) argues that economic capital often translates into social capital, providing greater opportunities for social interaction. Additionally, higher income allows women to engage in cultural activities, expanding their social networks (60). Moreover, the higher social status associated with wealth can help buffer the challenges posed by hypergamous marriages, facilitating smoother integration into diverse social circles. Thus, household income plays a crucial role in shaping social interactions in the context of educational disparities. This moderating effect aligns with global discussions on the intersection of socioeconomic status and social integration, suggesting that economic resources can act as a buffer to mitigate the negative social effects of educational inequalities.

Based on the study's findings, several targeted policies can help address the challenges of education-based hypergamy. First, promoting flexible work arrangements can alleviate the pressures of pursuing career aspirations, which limit social interactions. China has made strides in gender equality, with government initiatives such as paid maternity leave, the extension of flexible working hours, and support for remote work, particularly in urban centers (61, 62). These policies can be expanded to alleviate the social and professional pressures that women in education-based hypergamy face, enabling them to pursue career aspirations while maintaining a balanced social life. Additionally, economic empowerment programs that improve financial literacy and offer entrepreneurship opportunities can boost household income, helping to mitigate the negative effects of hypergamy (63). Programs aligned with China's “Common Prosperity” initiative, which seeks to reduce economic inequality, could play a significant role in empowering women to improve their financial standing and expand their social networks (64). Finally, social networking programs and gender equality campaigns can provide women with the skills and opportunities to build diverse social networks, fostering greater social integration (65). Policies aimed at improving women's participation in public life, including initiatives to support women's leadership roles and increase representation in the workplace, would also align well with these recommendations.

Despite its contributions, this study has several limitations. The cross-sectional nature of the data limits our ability to draw causal inferences. Future research should employ longitudinal designs to better understand the causal relationships between education-based hypergamy and social interaction. Moreover, the study relies on self-reported measures of social interaction, which may be subject to bias. Self-reported data may not always accurately reflect the frequency or quality of social interactions, and future studies could include objective measures, such as social network analysis or observational data, to strengthen the robustness of the findings. Another limitation is the reliance on Chinese regional data, which may limit the generalizability of the findings to other cultural contexts. Although the study highlights the experiences of women in education-based hypergamy in China, future research could explore the dynamics of hypergamy in other countries with differing cultural norms and economic structures. Furthermore, regional differences within China, particularly between more developed and less developed areas, could influence the degree to which hypergamy impacts social interaction. For example, women in more urbanized areas may experience different challenges and opportunities related to social engagement compared to those in rural regions.

In conclusion, our study underscores the importance of examining social interaction within the context of specific marital structures, such as education-based hypergamy. By highlighting the mediating roles of career aspirations and social distrust, as well as the moderating role of household income, the study provides a more comprehensive understanding of the relationship between education-based hypergamy and women's social interaction in China. These findings contribute to the existing literature on social capital, gender inequality, and public health, and offer practical implications for policy and future research. Addressing the challenges identified in this study can help enhance the wellbeing and social integration of women, ultimately contributing to a more inclusive and supportive society.

## Data Availability

The original contributions presented in the study are included in the article/supplementary material, further inquiries can be directed to the corresponding author.
